# Influence of Flour Particle Size Distribution on the Quality of Maize Gluten-Free Cookies

**DOI:** 10.3390/foods8020083

**Published:** 2019-02-23

**Authors:** Mayara Belorio, Marta Sahagún, Manuel Gómez

**Affiliations:** College of Agricultural Engineering, University of Valladolid, 34004 Palencia, Spain; msahaguncs@gmail.com (M.S.); pallares@iaf.uva.es (M.G.)

**Keywords:** maize flour, gluten-free, cookie, particle size, sieve

## Abstract

The objective of the present study was to analyse the influence of particle size distribution of maize flour in the formulation of gluten-free cookies. Different cookie formulations were made with three distinct maize flour fractions obtained by sieving (less than 80 µm; between 80 and 180 µm; greater than 180 µm). Cookies dimension, texture and colour were evaluated. Flour hydration properties and cookie dough rheology were also measured. Overall, an increase in maize flour particle size decreases the values of water holding capacity (WHC), swelling volume and *G*’ (elastic modulus) for the doughs. An increase in average particle size also increases diameter and spread factor of the cookies but decreases their hardness. A higher percentage of thick particles is more effective to reduce cookie hardness, but a certain percentage of thinner particles is necessary to give cohesion to the dough and to allow formation of the cookies without breaking. Cookies with a larger diameter also presented a darker colour after baking.

## 1. Introduction

Coeliac disease is an autoimmune disease related to an intolerance of gluten, which affects adults and children. It is treated by restricting gluten-containing food in the diet [1], which significantly increases the demand for gluten-free products. Therefore, to take advantage of this growth, many companies are looking to diversify and develop new products that meet this demand.

Most processed and pre-packaged bakery products, such as breads, cakes and cookies, are commonly produced with wheat flour. Although gluten plays an important role in bakery food processes, a glutenous structure is not developed in most types of cookies because of the high fat and sugar content in recipes and the scarce mechanical work imparted in the mixing process [2,3]. Thus, it is possible to obtain gluten-free cookies with characteristics very similar to those made with wheat flour [3,4,5]. Amongst cookies made with gluten-free flours, those formulated with maize flour are rated the highest by consumers [3].

In the case of cookies made with wheat flour, some characteristics, such as protein content or water absorption capacity, can justify its use in cookie preparation [2]. Maize, rice or legume flours are usually coarser than wheat flour due to their harder grains. It is already known that the particle size of wheat flour can influence cookie quality [6] but it could also be true for gluten-free flours. To investigate this, Rao et al. [7] observed that coarse sorghum flours produced cookies with lower hardness and better consumer acceptability. Mancebo et al. [8] also reported that coarse rice flours produced cookies with higher spread factors and lower hardness, which agrees with the observations by Ai et al. [9] with bean powders. However, Mancebo et al. [8] did not observe this effect in maize cookies, since both types of maize flour they used exhibited minor differences in particle size.

In some research papers, different particle size is obtained by forcing the grinding process until particles with a finer size are obtained [9,10]. However, this mechanical process causes an increase in damage starch that also affects how cookies develop during elaboration after elaboration [6,11]. There are few studies that achieve distinct fractions by sieving [3,7], nor are there any studies into blends of different flour fractions with different distributions of particle size. 

Thus, the present research proposes studying how different fractions of sieved white maize flour, both alone and in combination, could influence gluten-free cookie dough properties (hydration and rheology) and cookie quality (physical properties, texture and colour). 

## 2. Materials and Methods 

### 2.1. Cookie Ingredients

The white maize flour (5.87 g/100 g protein) used in this study was produced by Molendum Ingredients S.L. (Zamora, Spain). A Bühler MLI 300B sifter (Milan, Italy) holding 80 and 180 μm sieves was used for 15 minutes to obtain three maize flour fractions: A (<80 μm), B (80–180 μm) and C (>180 μm).

Other ingredients used in the cookie recipe were white sugar (AB Azucarera Iberia, Valladolid, Spain), margarine (Argenta crema, Puratos, Barcelona, Spain), sodium bicarbonate (Manuel Riesgo S. A., Madrid, Spain) and local tap water.

### 2.2. Flour Characterisation

Flour particle size was evaluated using a Mastersizer 3000 particle size analyser (Malvern Instruments, Malvern, UK). Values of D[4,3], which represents the equivalent spherical diameter of the particles, and of D(10), D(50) and D(90), which represent the maximum particle diameter below which 10%, 50% and 90% of the sample fall, respectively, were obtained. All the measurements were carried out in duplicate. 

Regarding hydration, water binding capacity (WBC, i.e., the amount of water retained by the sample after it has been centrifuged) was measured as described in AACC International method 56-30.01 [12]. Water holding capacity (WHC, i.e., the amount of water retained by the sample without being subjected to any stress) and swelling volume (SV, i.e., the volume occupied by a known weight of sample) were measured as described by Mancebo et al. [3]. All hydration properties were analysed in duplicate. 

### 2.3. Cookie Formulation

The original maize flour and the individual fractions were applied in different percentages giving rise to eleven combinations, as shown in Table 1. The mixing process was carried out as described by Mancebo et al. [8]. For the rheology test, the dough was rolled out to 3 mm thickness on a dough sheeter and then cut into round shape with a cutter of 60 mm diameter. For the baking test, samples were rolled out to 6 mm thickness and were cut with a 40 mm diameter cutter. The baking process was performed in a baking oven at 185 °C for 14 min. The cookies were cooled for one hour at room temperature and then placed in plastic bags, to avoid the interference of humidity. They were stored for seven days in a chamber with controlled temperature (25 °C) for further analysis. All cookie baking was done in duplicate.

### 2.4. Dough Rheology

Rheological behaviour of the fresh cookie dough was evaluated using a rheometer (Haake RheoStress 1, Thermo Fisher Scientific, Scheverte, Germany). Each dough sample was placed on a titanium parallel-serrated plate geometry PP60 Ti (60 mm diameter, 3 mm gap) and covered with Panreac Vaseline oil (Panreac Química S.A., Castellar del Vallés, Spain) to avoid drying during the test. A Phoenix II P1-C25P water bath maintained the temperature at 25 °C. 

In the first measurement, the cookie dough was subjected to a strain sweep (stress range of 0.1–100 Pa) at a constant temperature (25 °C) and frequency (1 Hz) to identify the linear viscoelastic region. Then, using these results, a stress value within the linear viscoelastic region was selected and applied in a frequency sweep test to obtain the values of the elastic modulus (*G*’, Pa), viscous modulus (*G*”, Pa), complex modulus (G*) and tan delta (*G*”/*G*’) over a range of frequency values (*w*, Hz). The measurements were made in duplicate.

### 2.5. Cookie Characteristics 

Cookie moisture was evaluated as described by AACC method 44-15.02 [13] and the test was made in duplicate.

Cookie diameter was measured twice, in perpendicular directions, to achieve an average diameter (D). Cookie thickness (T) was also measured to obtain the spread factor (D/T). 

Texture parameters of the cookies were analysed by using a Texture TA-XT2 texture analyser (Stable Micro Systems, Surrey, UK). The peak force, or hardness, (N) and the elastic modulus (N/mm^2^) were obtained by the compression of a ‘three-point bending’ test with a three-point bending rig probe (HDP/3PB). The measurement conditions were: travel distance of 20 mm, trigger force of 5 g and test speed of 2.0 mm/s. 

Cookie colour was measured at the centre of the surface crust with a Minolta CN-508i spectrophotometer (Minolta Co. Ltd., Osaka, Japan) using a D65 illuminant with a 2° standard observer angle. L*, a* and b* values were expressed in the colour space defined by the International Commission on Illuminaion (CIE).

All cookie characteristics were measured in six cookies of each batch, seven days after baking.

### 2.6. Statistical Analysis 

Statistical analysis of the differences between the parameters of the different formulations were evaluated by analysis of variance (ANOVA) using Statgraphics Centurion XVI software (StatPoint Technologies Inc., Warrenton, DC, USA). Fisher’s least significant difference (LSD) was used to describe means with 95% confidence intervals.

## 3. Results

### 3.1. Particle Size and Hydration Properties

As shown in Table 2, the different blends presented an average size D[4,3] within the range of particle sizes of the fractions that comprised it. As expected, the average particle size of blends formed from fractions A and B increased with the quantity of fraction B. This also occurred with the mixtures of fractions A and C. The control sample had an average particle size slightly higher than fraction B, but clearly lower than fraction C. D(10) and D(50) values decreased as the percentage of fraction A increased. However, for mixtures containing 50% or more of A, there were no further significant changes, regardless of whether they were mixed with B or C. This is logical if it is remembered that these values refer to 10% or 50% of the thinnest flours from the mixtures obtained from fraction A. In the case of mixtures with 25% of A, there was a significant difference in D(50) values and they were even smaller in mixtures with the fraction B. Control flour had relatively low D(10), which was similar to mixtures with 25% of A and smaller than mixtures without fraction A. For its part, the D(50) is minor in all mixtures with A, with just one exception (25A/75C), and no significant differences were observed with fraction B. In general, the average sizes of the flours used in this research were similar to those applied in other studies on gluten-free cookie formulations [3,4,7].

According to Table 3, higher values of WHC are related to the compositions A and B, while lower values are found in mixtures with percentages of C higher than 50% (100C, 25A/75C and 50A/50C). In fact, there is a negative correlation between the WHC and D[4,3] *r* = −0.74 (significant at 99%). The relationship between WHC and the particle size was observed by other authors [10] and can be explained by the large surface area presented by finer flours. 

Nevertheless, other studies were either not able to establish a correlation between particle size and WHC, or no decrease in WHC was found with reductions in particle size [14]. These differences could be related to both different compositions of particles with distinct sizes and to the morphology and particle size distribution, which was not evaluated in other studies. In this case, it is important to stress that the correlation of WHC with D(90) values was higher than with D[4,3], *r* = −0.86 (significant at 99.9%). This fact may indicate that the percentage of coarse fractions is what most affects this property. This could be explained by the fact that the finer fractions present similar particle sizes while the thicker fractions have greater differences between them. The swelling volume is highly correlated with WHC, D[4,3], and D(90) (*r* = 0.80, significant at 99%). Nevertheless, no correlation between particle size and WBC values was found, as observed by De la Hera et al. [15]. Thus, the larger values of WBC are related with fraction B followed by other individual fractions (pure A and pure C) and mixtures with the coarser fractions (B/C). On the other hand, the smaller values of WBC were observed in mixtures comprising A and B, except 50A/50B, which showed no significant difference compared to the control. Combinations of A and C presented intermediate values with no significant differences. Intermediate values of WBC were also shown by the control flour. In this case, the percentage of different fractions, their packing capacity and particle morphology seems to have a higher influence on this behaviour than the average particle size when using fine and coarse flours together in the centrifugal process.

### 3.2. Dough Rheology Properties

Dough rheology is a fundamental characteristic for cookie formulation so that if the dough is very soft or firm it is not easy to manipulate. The dough must be sufficiently cohesive to remain united during the different phases of processing and to be easily cut by the moulds [16]. In fact, an important finding from our rheology measurements was that values were obtained only for samples containing fraction A. The other doughs were extremely brittle and were impossible to laminate without breaking. From this result, it is possible to conclude that to make a cookie dough that is cohesive and laminable, it is necessary for it to contain a minimum proportion of reduced size particles. This conclusion was not found in other studies because those only considered pure fractions with a unique size, bigger or smaller, obtained by screening or by another grinding process. 

In general, *G*’ values were higher than *G*” values (Table 3), showing that the elastic component is dominant over the viscous one, which suggests a solid elastic-like behaviour of all the doughs studied. This result was confirmed in values of tan delta that were all lower than 1.0, in agreement with other studies about cookies [8,17,18]. Between different doughs, smaller values of *G*’ corresponded to those flours with a higher value of D[4,3], such as the control flour and the mixture 25A/75B. Meanwhile, the biggest values of *G*’ were found in doughs with finer flours (with lower D[4,3]) with a higher percentage of fraction A. This corresponded with low values of D(10) (100A, 75A/25B, 75A/25C). This indicates that finer flours have a better packing quality, which results in more cohesive doughs. However, it is also important to note the larger values of *G*’ obtained with mixtures of fractions A and C where C made up more than 50%. In those cases, it was very difficult to manipulate the doughs because of their weakness. These samples presented a different texture compared with other samples because they showed an anomalous rheological behaviour. These doughs were like those where it was not possible to measure the rheology parameters, but their breakability characteristics were less extreme. 

Regarding the viscous component (*G*”), it was not possible to find significant differences between the samples. Probably, this fact could be due to the excessive variability of the data, especially of the more fragile doughs. The same effect was observed in tan delta values, where some differences were found between control flour and the 25A/75C mixture, which were the most difficult samples to perform the rheology test on because of the cohesiveness. Even though previous studies reported correlations between rheological cookie values and hydration properties when flours or mixtures were used [18,19,20], in this case, no correlations were observed. This was due to the anomalous brittleness of samples with smaller proportions of fraction A and the importance between the particle size distribution and rheological behaviour.

### 3.3. Cookie Characteristics

**Figure 1:**
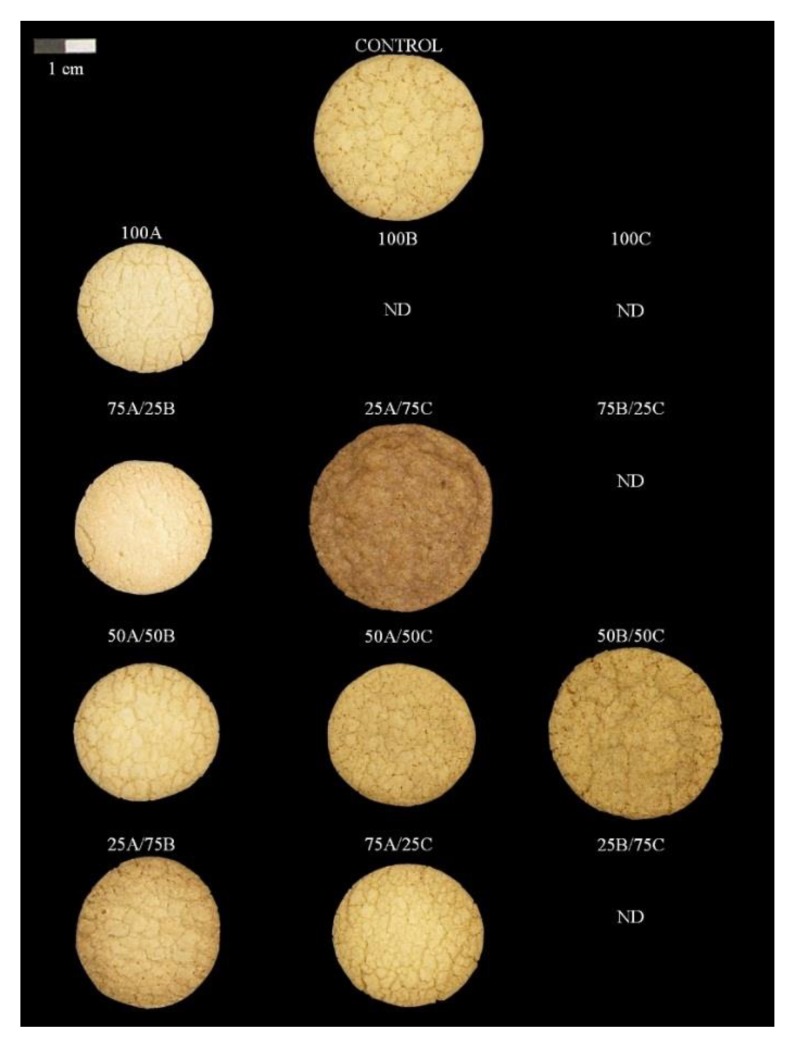
Image from cookies made with different particles sizes of white maize flour.

Cookie characteristics are shown in Table 4. It is important to underline that it was not possible to make cookies with pure fractions B and C because the dough was very breakable (Figure 1). This brittle character was also observed in B/C mixtures (25B/75C, 50B/50C and 75B/25C) where, even though it was not possible to measure the rheological parameters, only the mixture 50B/50C was at least capable of being made. This fact could be explained because the rheological test required a thin layer of the dough, which was easily broken. These three flours (B, C, and B/C) were the ones that presented higher values of D(10), which means that it is necessary to add a small number of finer particles to the fractions. These particles take their place between the thick particles and increase the cohesiveness of the dough which allows the dough to be laminated. Furthermore, a mixture made using different particle sizes (B/C) seems to be more convenient than those made of only one size, which can be explained by the same effect of fine particles getting placed between coarse ones. 

Among the obtained cookies, no significant differences were found in final product moisture, and in all cases the values were less than 1%. It is also important to stress that the 25A/75C cookies showed the highest diameter and spread factor followed by those made with 50% of C (A/C and B/C). This fact seems to indicate that a higher percentage of coarse flour is favourable to releasing and spreading during baking, generating cookies of a greater diameter. This was also observed by Mancebo et al. [3] with rice flours, Rao et al. [7] with sorghum flour and Ai et al. [9] with dry bean powders. For their part, fractions with a higher percentage of A and, therefore, a lower average size (A, 75A/25B and 75A/25C) showed small spread factors; the next lowest spread factor was 50A/50B (with larger average size). In fact, 99.9% significant correlations were found between the diameter and D[4,3] (*r* = 0.98) and between the spread factor and D[4,3] (*r* = 0.92). Some authors affirm that flours with a lower hydration capacity produced cookies with higher diameters because they allowed excess water to dissolve the sugar, to reduce the initial viscosity of the doughs and to allow more expansion during baking [21,22]. Thus, in this study, significant correlations of 99% were found between WHC and the values of diameter (*r* = −0.74) and spread factor (*r* = −0.72), as found in similar studies [3,8]. In this way, it seems that the value of D[4,3] is a better indicator of dough expansion during baking and the final diameter, but D(10) shows a better possibility of obtaining cohesive doughs that can be laminated and cut without breaking. 

Regarding the texture of the cookies, a significant correlation coefficient of *r* = 0.88 was found in all cases but at differing significance levels: 99.9% significance between hardness, diameter and spread factor; 99% between the elastic modulus and diameter and 95% between the elastic modulus and the spread factor). Therefore, cookie dimensions showed a stronger correlation with hardness than with the gradient obtained by the curve. Thus, harder cookies presented both a lower diameter and spread factor (100A and 75A/25B). On the other hand, less hard cookies showed higher spread factors (25A/75C) followed by those mixtures with A/C and B/C which presented values greater than all the other samples. Mancebo et al. [3] described how cookies with higher spread factors showed a lower peak force in textural analysis, but we found no correlation whatsoever between these two parameters for any of the cookies we analysed. In addition to the dimensions of the cookies, these differences may be related to their internal structure, the particle size of the flours and their compaction capacity. Parameters of texture and indicators of flour particle size showed a better correlation than with cookie dimensions, which demonstrates that as the particle size decreases, the hardness of the cookies increases, in agreement with the observations of similar studies [7,9]. In the case of D[4,3], the correlation with hardness also presented a significant correlation of *r* = −0.87 (99% significance). One observed correlation, between hardness and D(90), stands out (*r* = −0.93, significant at 99.9%), as it is higher even than the ones observed between hardness and D(50) (*r* = −0.72, significant at 99%) and between hardness and D(10) (*r* = −0.61, significant at 95%). Therefore, it seems that what most reduced the hardness of the cookies was an increase in the percentage of thicker particles, even though it is necessary to remember the importance of having a certain percentage of smaller particles to allow the formation of the cookies.

Regarding colour, important differences were found among the values of L*. Cookies with a larger percentage of A (100% or 75%) were lighter in colour and presented lower spread factors. Meanwhile, cookies made with a larger percentage of C (25A/75C) showed darker colour (small values of L*) followed by those made with 50% of C. With regard to a* and b* values, only those samples with extreme mixes stand out: sample A, which had a lower particle size, diameter and spread factor, presented the smallest values of a*, while the sample 25A/75C, which had a higher particle size, spread factor and diameter, showed the highest values of a* and smallest of b*. Mancebo et al. [3] also observed the smallest values of L* and b* for cookies with higher spread. This could be because of a higher diameter and a lower thickness of cookies, which produces high temperatures in a short time within the dough. Thus, temperature increases led to more caramelization and Maillard reactions, which are the main causes of final cookie colour [23]. In fact, significant correlations of 99.9% were found between the spread factor of the cookies and the parameters L* (*r* = −0.91), a* (*r* = −0.87) and b* (*r* = −0.81).

## 4. Conclusions

The final characteristics of cookies can be influenced by the properties of the ingredients used in their elaboration such as the particle size of the flour, the sugar content and the fat used in formulation. There are no references about how both of these last factors can affect the quality of the cookies, which may be an opportunity for future studies. However, previous studies proved that a higher average particle size favours spread factor and decreases cookie hardness. Nevertheless, this study proves for the first time that it is necessary to also have a certain percentage of fine particles, which, being placed between coarser particles in the dough, give rise to a higher dough cohesiveness. Otherwise, doughs are excessively fragile in the laminating process and cookies are impossible to be made. These results must be considered in future studies about gluten-free cookies and in food development in the industry.

## Figures and Tables

**Table 1:** Gluten-free cookie formulations presented in grams with different percentages of maize flour and fractions A (<80 μm), B (80–180 μm) and C (>180 μm).

Ingredients	CF	100A	100B	100C	50A/50B	50A/50C	50B/50C	25A/75B	75A/25B	25A/75C	75A/25C
White maize flour	173.2	-	-	-	-	-	-	-	-	-	-
A (<80 μm)	-	173.2	-		86.6	86.6	-	43.3	129.9	43.3	129.9
B (80–180 μm)	-		173.2	-	86.6	-	86.6	129.9	43.3	-	-
C (>180 μm)	-		-	173.2	-	86.6	86.6	-	-	129.9	43.3
White sugar	124.8	124.8	124.8	124.8	124.8	124.8	124.8	124.8	124.8	124.8	124.8
Margarine	77.6	77.6	77.6	77.6	77.6	77.6	77.6	77.6	77.6	77.6	77.6
Sodium bicarbonate	3.6	3.6	3.6	3.6	3.6	3.6	3.6	3.6	3.6	3.6	3.6
Tap water	25.0	25.0	25.0	25.0	25.0	25.0	25.0	25.0	25.0	25.0	25.0

CF: control formulation.

**Table 2:** Maize flour, maize flour fractions and their combinations particle size measurements.

	D[4,3]	D(10)	D(50)	D(90)
Control flour	199.9 ± 6.4 ^g^	26.5 ± 1.4 ^c^	178.8 ± 6.5 ^e^	401.4 ± 8.8 ^e^
100A (<80 μm)	61.6 ± 3.9 ^a^	14.6 ± 0.4 ^a^	49.4 ± 4.7 ^a^	128.3 ± 7.2 ^a^
100B (80–180 μm)	186.3 ± 9.2 ^f^	88.3 ± 9.6 ^d^	177.0 ± 8.5 ^e^	304.7 ± 9.7 ^d^
100C (>180 μm)	354.2 ± 5.4 ^i^	211.2 ± 1.4 ^f^	337.4 ± 4.5 ^g^	533.6 ± 12.2 ^h^
50A/50B	124.1 ± 1.3 ^c^	19.1 ± 0.4 ^ab^	105.0 ± 0.6 ^c^	263.2 ± 4.2 ^c^
50A/50C	172.2 ± 4.8 ^e^	18.9 ± 0.3 ^ab^	105.6 ± 4.5 ^c^	415.9 ± 8.6 ^e^
50B/50C	261.1 ± 3.8 ^h^	113.1 ± 3.0 ^e^	242.4 ± 3.7 ^f^	444.7 ± 4.9 ^f^
25A/75B	151.9 ± 3.8 ^d^	27.7 ± 1.3 ^bc^	144.0 ± 5.4 ^d^	287.3 ± 2.7 ^cd^
75A/25B	101.1 ± 10.0 ^b^	16.9 ± 0.4 ^a^	74.2 ± 8.2 ^b^	230.7 ± 23.3 ^b^
25A/75C	252.8 ± 3.8 ^h^	27.7 ± 0.1 ^c^	250.8 ± 3.4 ^f^	489.8 ± 9.0 ^g^
75A/25C	113.1±4.4 ^bc^	16.5 ± 0.2 ^a^	66.5 ± 1.6 ^b^	299.5 ± 15.4 ^d^

D[4,3]: average particle size which constitutes the bulk of the sample volume. D(10): maximum particle diameter below which 10% of the sample falls. D(50): maximum particle diameter below which 50% of the sample falls. D(90): maximum particle diameter below which 90% of the sample falls. Data are expressed as means ± standard deviation (SD) of duplicate assays. The values with the same letter in the same column do not present significant differences (at a significant level of *p* < 0.05).

**Table 3:** Maize flour, maize flour fractions and their combinations: hydration properties and cookie dough rheology.

	Hydration Properties			Dough Rheology		
	WHC	Swelling	WBC	*G*’ (×10^6^)	*G*” (×10^6^)	Tan Delta
Control flour	1.57 ± 0.04 ^bcd^	2.09 ± 0.16 ^abc^	1.28 ± 0.04 ^de^	0.42 ± 0.02 ^a^	0.25 ± 0.27 ^a^	0.59 ± 0.54 ^b^
100A (<80 μm)	1.85 ± 0.07 ^fg^	2.29 ± 0.16 ^d^	1.30 ± 0.01 ^ef^	1.36 ± 0.27 ^c^	0.26 ± 0.18 ^a^	0.15 ± 0.04 ^ab^
100B (80–180 μm)	1.87 ± 0.13 ^g^	2.19 ± 0.01 ^bcd^	1.47 ± 0.01 ^g^	ND	ND	ND
100C (>180 μm)	1.48 ± 0.03 ^bc^	2.20 ± 0.00 ^cd^	1.30 ± 0.01 ^ef^	ND	ND	ND
50A/50B	1.64 ± 0.10 ^cde^	2.09 ± 0.14 ^abc^	1.27 ± 0.04 ^de^	0.72 ± 0.02 ^ab^	0.12 ± 0.17 ^a^	0.24 ± 0.35 ^ab^
50A/50C	1.43 ± 0.09 ^ab^	1.99 ± 0.01 ^a^	1.19 ± 0.01 ^bc^	1.20 ± 0.22 ^c^	0.10 ± 0.13 ^a^	0.10 ± 0.14 ^ab^
50B/50C	1.83 ± 0.05 ^fg^	2.27 ± 0.11 ^cd^	1.33 ± 0.01 ^f^	ND	ND	ND
25A/75B	1.74 ± 0.11 ^efg^	2.18 ± 0.01 ^abcd^	1.15 ± 0.02 ^b^	0.27 ± 0.23 ^a^	0.05 ± 0.30 ^a^	0.25 ± 0.11 ^ab^
75A/25B	1.69 ± 0.04 ^def^	2.19 ± 0.02 ^bcd^	1.05 ± 0.01 ^a^	1.25 ±0.18 ^c^	0.06 ± 0.88 ^a^	0.05 ± 0.08 ^ab^
25A/75C	1.41 ± 0.02 ^a^	2.00 ± 0.01 ^ab^	1.20 ± 0.01 ^c^	1.49 ± 0.17 ^c^	0.08 ± 0.11 ^a^	0.01 ± 0.00 ^a^
75A/25C	1.56 ± 0.04 ^abcd^	2.20 ± 0.01 ^cd^	1.24 ± 0.04 ^cd^	1.05 ± 0.15 ^bc^	0.06 ± 0.90 ^a^	0.14 ± 0.01 ^ab^

ND: no development. WHC: water holding capacity. WBC: water binding capacity. Data are expressed as means ± SD of duplicate assays. The values with the same letter in the same column do not present significant differences (at a significance level of *p* < 0.05).

**Table 4:** Physical properties of cookies.

	Dimensions		Texture		Colour		
	Diameter (mm)	Spread Factor	Hardness (N)	Cookie Elastic Modulus (N/mm²)	L*	a*	b*
Control flour	56.4 ± 0.40 ^c^	7.48 ± 0.31 ^de^	34.10 ± 1.22 ^cd^	26.90 ± 1.52 ^ab^	55.33 ± 1.97 ^c^	5.59 ± 2.79 ^abc^	18.33 ± 0.92 ^b^
100A (<80 μm)	43.1 ± 1.22 ^a^	4.05 ± 0.24 ^a^	50.84 ± 1.97 ^f^	54.48 ± 2.73 ^d^	78.45 ± 0.95 ^f^	3.70 ± 1.40 ^a^	21.41 ± 1.02 ^bcd^
100B (80–180 μm)	ND	ND	ND	ND	ND	ND	ND
100C (>180 μm)	ND	ND	ND	ND	ND	ND	ND
50A/50B	48.4 ± 0.22 ^bc^	5.54 ± 0.34 ^bc^	34.43 ± 1.94 ^cd^	33.41 ± 3.13 ^bc^	69.44 ± 1.27 ^e^	5.47 ± 1.12 ^abc^	21.37 ± 0.40 ^bcd^
50A/50C	55.1 ± 0.20 ^d^	8.47 ± 0.20 ^ef^	24.25 ± 0.37 ^b^	27.15 ± 3.97 ^ab^	61.20 ± 0.11 ^d^	7.19 ± 0.76 ^bcd^	19.14 ± 1.04 ^b^
50B/50C	54.9 ± 2.53 ^d^	9.47 ± 1.02 ^f^	25.98 ± 0.30 ^b^	19.83 ± 0.45 ^a^	51.11 ± 1.23 ^b^	7.85 ± 1.40 ^cd^	13.37 ± 1.04 ^a^
25A/75B	50.7 ± 0.07 ^c^	6.31 ± 0.13 ^cd^	36.24 ± 0.05 ^d^	38.54 ± 1.38 ^c^	69.84 ± 1.89 ^e^	5.98 ± 0.55 ^abc^	25.23 ± 1.12 ^d^
75A/25B	43.9 ± 1.22 ^a^	4.53 ± 0.19 ^ab^	42.82 ± 1.98 ^e^	38.71 ± 10.61 ^c^	75.66 ± 1.80 ^f^	4.68 ± 0.60 ^ab^	24.42 ± 1.32 ^cd^
25A/75C	63.0 ± 0.71 ^e^	14.01 ± 1.31 ^g^	17.93 ± 0.8 ^a^	29.70 ± 5.37 ^abc^	46.52 ± 3.10 ^a^	9.71 ± 1.00 ^d^	12.93 ± 2.13 ^a^
75A/25C	47.14 ± 0.79 ^b^	5.10 ± 0.01 ^abc^	33.24 ± 0.80 ^c^	34.81 ± 1.74 ^bc^	74.34 ± 2.63 ^f^	5.19 ± 0.08 ^abc^	21.06 ± 4.24 ^bc^

ND: no development. Data are expressed as means ± SD of duplicate assays. The values with the same letter in the same column do not present significant differences (at a significance level of *p* < 0.05).
